# Sinusoidal Obstruction Syndrome With Inotuzumab Ozogamicin Combined With Ponatinib for Philadelphia‐Chromosome‐Positive Lymphoblastic Malignancies

**DOI:** 10.1002/jha2.70339

**Published:** 2026-07-02

**Authors:** Emi Yokoyama, Satoshi Ota, Yasuo Sakurai, Toma Suzuki, Naoki Miyashita, Minoru Kanaya, Koh Izumiyama, Makoto Saito, Masanobu Morioka, Akio Mori, Takeshi Kondo

**Affiliations:** ^1^ Blood Disorders Center Aiiku Hospital Sapporo Japan; ^2^ Department of Pathology Teine Keijinkai Hospital Sapporo Japan; ^3^ Department of Radiology Teine Keijinkai Hospital Sapporo Japan; ^4^ Department of Diagnostic and Interventional Radiology Sapporo Higashi Tokushukai Hospital Sapporo Japan; ^5^ Department of Hematology Hakodate Municipal Hospital Hakodate Japan; ^6^ Department of Hematology Asahikawa City Hospital Asahikawa Japan

**Keywords:** inotuzumab ozogamicin, Philadelphia‐chromosome‐positive lymphoid malignancies, ponatinib, sinusoidal obstruction syndrome, veno‐occlusive disease

## Abstract

**Introduction:**

Sinusoidal obstruction syndrome, also known as veno‐occlusive disease (SOS/VOD), can occur after exposure to inotuzumab ozogamicin (InO), a calicheamicin‐based antibody‐drug conjugate.

**Case Presentation:**

We experienced two cases of SOS/VOD after treatment with InO combined with intermittent administration of ponatinib without hematopoietic stem cell transplantation for Philadelphia‐chromosome‐positive (Ph+) lymphoblastic malignancies. Both cases developed SOS/VOD after two courses of InO. Ultrasound scoring system and pathological assessment were useful for diagnosis of SOS/VOD.

**Conclusion:**

This is the first report warning of the potentially high risk of developing SOS/VOD with the combination of InO and ponatinib, both of which can induce endothelial cell damage.

**Trial Registration:**

The authors have confirmed clinical trial registration is not needed for this submission

## 1 Introduction

Sinusoidal obstruction syndrome, also known as veno‐occlusive disease (SOS/VOD), is a potentially life‐threatening complication observed mainly after hematopoietic stem cell transplantation (HSCT). It can occur after exposure to radiation, several cytotoxic agents, calicheamicin‐based antibody‐drug conjugates, immunosuppressants, and specific plants. The EBMT 2023 criteria are a newly refined diagnostic classification of SOS/VOD, replacing the conventionally used Baltimore or modified Seattle criteria (Table 1) [1]. Unlike the conventional criteria, the presence of hyperbilirubinemia and jaundice is not necessarily required in the EBMT 2023 criteria. It classifies adults SOS/VOD into “probable,” “clinical,” and “proven” SOS/VOD. In this classification, ultrasound (HokUS‐10) (Table 2) and/or elastography findings are also considered to be helpful in diagnosing SOS/VOD.

**TABLE 1:** EBMT 2023 criteria of SOS/VOD.

**Probable**	**Clinical**	**Proven**
Two of the following criteria must be present:	Bilirubin ≥ 2 mg/dL and two of the following criteria must be present:	Historically proven SOS/VOD or hemodynamically proven (HVPG ≥ 10 mmHg)
Bilirubin ≥ 2 mg/dL		
Painful hepatomegaly	Painful hepatomegaly	
Weight gain > 5%	Weight gain > 5%	
Ascites	Ascites	
Ultrasound and/or elastography are suggestive of		
SOS/VOD		
**Onset**		
In the first 21 days after HSCT: classical SOS/VOD		
> 21 days after HSCT: late onset SOS/VOD		

Abbreviation: HVPG, hepatic venous pressure gradient.

**TABLE 2:** HokUS‐10 scoring.

**Parameters**		**Points**
Hepatic left lobe vertical diameter	≥ 70 mm	1
Hepatic right lobe vertical diameter	≥ 110 mm	1
Gallbladder wall thickening	≥ 6 mm	1
PV diameter	≥ 12 mm	1
PUV diameter	≥ 2 mm	2
Amount of ascites	Mild	1
	Moderate to severe	2
PV mean velocity	< 10 cm/s	1
Direction of PV flow	Congestion or hepatofugal	1
Appearance of PUV blood flow signal	Yes	2
Hepatic artery RI	≥ 0.75	1

Abbreviations: PV, portal vein; PUV, peri‐umbilical vein; RI, resistive index.

Inotuzumab ozogamicin (InO) is a calicheamicin‐conjugated CD22 antibody that is effective against refractory or relapsed Philadelphia‐chromosome‐positive (Ph+) acute lymphoblastic leukemia (ALL) [2, 3]. The mechanism by which InO induces SOS/VOD is postulated to be due to the non‐specific uptake of InO by liver sinusoidal endothelial cells, since the CD22 expression is absent in the normal liver [4, 5]. Tyrosine kinase inhibitors (TKIs) are essential components of treatment strategy in Ph+ ALL. Among TKIs, ponatinib is especially highly effective in controlling the disease with or without T315I mutation.

We report two cases of SOS/VOD without HSCT after treatment with InO combined with ponatinib for Ph+ lymphoblastic malignancies.

## 2 Case Presentation

### 2.1 Case 1

**FIGURE 1:**
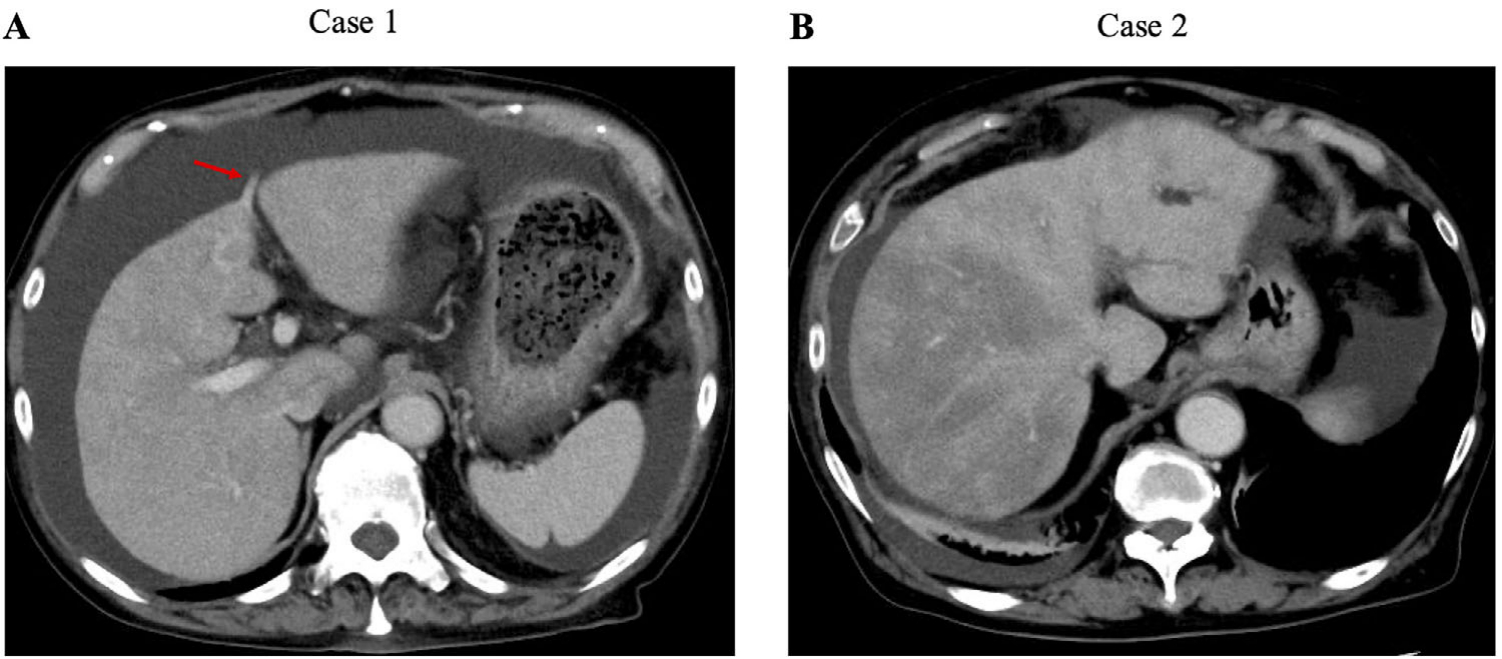
Radiological and pathological findings of the two cases. (A) Abdominal CT in Case 1 showed moderate to severe ascites and a patent umbilical vein (red arrow). (B) Abdominal CT in Case 2 showed mild ascites and a mosaic pattern of enhancement.

An 85‐year‐old female was diagnosed with chronic myeloid leukemia in lymphoid blast crisis (CML‐BC). After reduced‐dose cyclophosphamide, daunorubicin, vincristine, and prednisolone (CHOP) therapy, ponatinib (30 mg QD) was initiated and continued, which induced a deep molecular response. At 2.5 years after the onset, the dose of ponatinib was reduced to 15 mg QD, considering the risk of cardiovascular events. At 1.5 years after ponatinib dose reduction, hematological recurrence of the disease was observed, and InO was initiated (28‐day cycle, Cycle 1: Day 1, 0.8 mg/m^2^; Day 8 and 15, 0.5 mg/m^2^; Cycle 2: Day 1, 8, and 15, 0.5 mg/m^2^). Ponatinib was continued with the increased dose of 30 mg QD with the expectation of disease control. Ponatinib was interrupted on the days of InO treatment because concomitant administration of these drugs is off‐label. After one cycle of InO, the international scale (IS) of major *BCR::ABL1* became undetectable. Treatment with InO was continued to Day 1 of Cycle 3, and ponatinib (30 mg QD) monotherapy was continued thereafter. Around that time, ascites and systemic edema developed. She gained 8.8 kg (14.5% increase) in body weight. Laboratory data showed a slight elevation in aminotransferases and C‐reactive protein (CRP). No painful hepatomegaly or hyperbilirubinemia was observed. Computed tomography (CT) showed moderate‐to‐severe ascites and a patent umbilical vein (Figure 1A). The maximum HokUS‐10 score was 6 (moderate to severe ascites, peri‐umbilical vein diameter 2.3 mm, portal vein mean velocity 6.7 cm/s, and hepatic artery resistive index 0.85). Based on the clinical symptoms and US findings, she was diagnosed with probable SOS/VOD after 2 months from the last InO administration. Treatment with ponatinib was discontinued, and diuretics (furosemide and tolvaptan) and corticosteroids (prednisolone 0.5 mg/kg) were initiated. Fortunately, the IS of the major *BCR::ABL1* was below the detection level for several months without treatment for CML‐BC. The symptoms of SOS/VOD gradually improved, and ponatinib (15 mg QD) was restarted 8 months after the diagnosis of SOS/VOD.

### 2.2 Case 2

**FIGURE 2:**
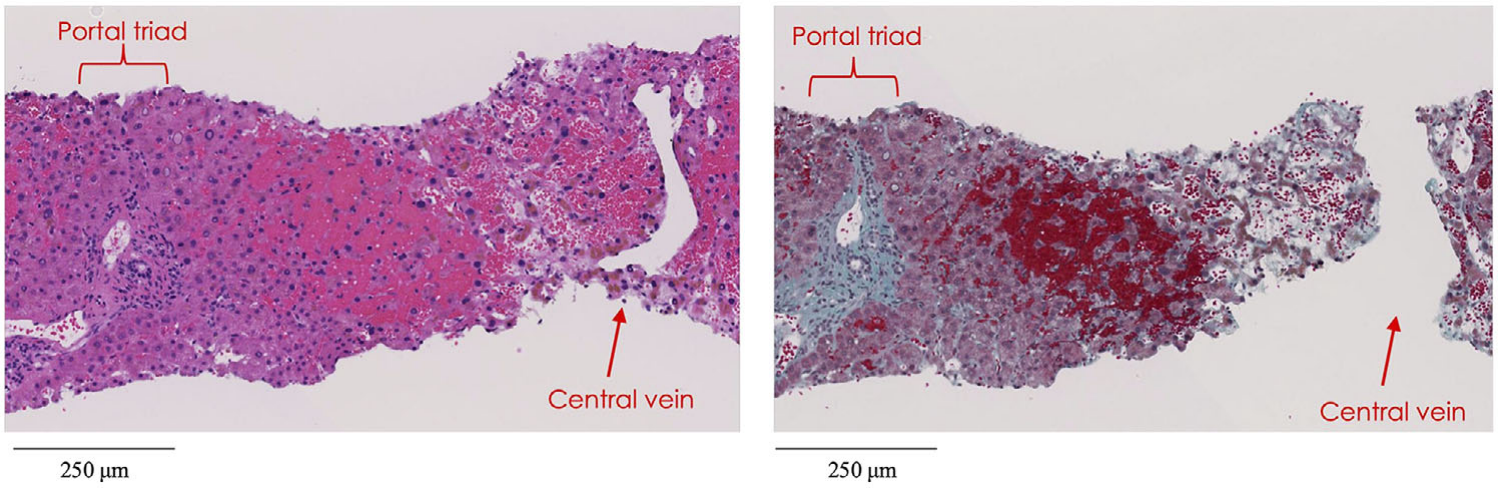
Histopathology of Case 2. Left: Hematoxylin‐eosin staining showing hepatocyte degeneration and marked congestion with infiltration of lymphocytes and hemosiderin‐laden macrophages. Right: Elastica‐Masson stain showing periportal vein fibrosis.

An 82‐year‐old male was diagnosed with Ph+ ALL. After induction therapy with dasatinib and prednisolone, the patient achieved a hematological response, but measurable residual disease (MRD) with minor *BCR::ABL1* was positive. Blinatumomab (42‐day cycle, Cycle 1: Days 1–7, 9 µg; Day 8–28, 28 µg) was initiated, and ponatinib (15 mg QD) was administered from Day 29 to Day 42. Blinatumomab was continued for three cycles, but the MRD level gradually increased. Treatment was switched to InO, and ponatinib (15 mg QD) was continued with the same administration schedule as that described in Case 1. After seven doses of InO (Cycle 3, Day 1), ascites and systemic edema developed. The treatment with InO and ponatinib was discontinued. He gained 6.2 kg (+11%) in body weight, and slight elevations of aminotransferases and CRP, like Case 1, were observed. He did not show either painful hepatomegaly or hyperbilirubinemia. CT showed mild ascites and a mosaic pattern of enhancement (Figure 1B). The maximum HokUS‐10 score was 3 (moderate ascites, hepatic right lobe diameter 125 mm). Under suspicion of SOS/VOD, the patient underwent transjugular liver biopsy. Pericentral and periportal vein fibrosis, hepatocyte degeneration, and marked congestion with infiltration of lymphocytes and hemosiderin‐laden macrophages were observed, consistent with the pathological findings of SOS/VOD (Figure 2). Based on the histological findings, the patient was diagnosed with proven SOS/VOD. Defibrotide (6.25 mg/kg Q6H) was initiated and continued for 5 weeks along with diuretics (furosemide). The patient's symptoms improved slowly. During this period, the MRD of Ph+ ALL was detectable and showed slight elevation. Ponatinib (15 mg QD) was carefully restarted 3 months after the diagnosis of SOS/VOD.

## 3 Discussion

Diagnosing SOS/VOD using conventional diagnostic criteria was challenging in our cases, as they did not show any significant elevation of total bilirubin or painful hepatomegaly, both of which are typical features of SOS/VOD following HSCT. HokUS‐10 is a newly proposed scoring system with US findings demonstrating a high sensitivity and specificity (100% and 95.8%, respectively), with a cut‐off value of 5 in cases following HSCT [6]. Based on the US findings along with the clinical symptoms, Case 1 was diagnosed as probable SOS/VOD. A liver mosaic pattern of enhancement on CT has been reported when sinusoidal dilatation occurs with SOS [7]. Case 2 showed typical findings on a CT scan, which suggested the diagnosis of SOS. The reported histopathologic features of SOS/VOD include sinusoidal dilatation, atrophy, or the dissociation of hepatic plates, central venous and perisinusoidal fibrosis, centrilobular hemorrhage, and nodular degeneration predominantly observed within the centrilobular or midzonal regions [8]. In Case 2, the liver biopsy contributed to the diagnosis of proven SOS/VOD. A liver biopsy is usually not feasible percutaneously in the presence of ascites and low platelet counts, as observed in SOS/VOD. A transjugular biopsy is a safe method for diagnosing SOS/VOD in such cases, if available. Liver stiffness measurements (LSMs) are also reported to contribute to differential diagnosis and to treatment response of SOS/VOD [9], and are included in the EBMT 2023 criteria, although we did not evaluate LSM in these two patients. The EBMT 2023 criteria seem to be useful for diagnosing SOS/VOD without HSCT as observed in our cases.

In the Phase 3 INO‐VATE trial, the median treatment duration and the number of cycles for the InO arm were 8.9 weeks and three cycles, respectively [10]. The incidence of SOS/VOD in InO arm, which did not receive HSCT after InO treatment, was only 3.0%. The cumulative dose of InO in our cases was not high compared to that in the INO‐VATE study. We speculate that the combination of InO and TKIs may augment the risk of SOS/VOD. In the interim results of a Phase II study investigating dasatinib and InO‐based induction for newly diagnosed Ph+ ALL, the scheme was amended during the study to administer dasatinib sequentially after InO rather than concomitantly due to the occurrence of SOS/VOD [11]. On the other hand, in the Phase 1/2 study, which assessed the combination therapy with InO and bosutinib for relapsed or refractory Ph+ ALL, there were no cases of SOS/VOD even though the two agents were administered concomitantly [12]. Among TKIs, dasatinib, ponatinib, and nilotinib have been reported to have differential toxic effects on endothelial cells, including impaired wound healing, survival, proliferation, cell migration, and cell junctional integrity [13]. Considering the toxic impact of InO on endothelial cells, a combination of InO and these TKIs may pose a high risk of damaging sinusoidal endothelial cells in the liver. The onset of SOS/VOD occurred after two courses of InO in both cases, thus indicating the cumulative endothelial cell toxicity of InO, especially when combined with ponatinib. It might have been appropriate to discontinue InO earlier, especially after we confirmed MRD negativity in Case 1. Although the severity of SOS/VOD in our cases was moderate and not life‐threatening, it took a long time to recover sufficiently to be able to resume treatment for the malignancy.

The limitation of this report is the small number of cases for generalized interpretation. Furthermore, we cannot exclude the possibility that SOS/VOD was caused by InO itself and was independent of ponatinib.

## 4 Conclusions

InO and ponatinib are important treatment options for refractory and relapsed Ph+ lymphoblastic malignancies. However, the treatment duration and combination should be carefully considered to avoid synergistic toxicity. The combination of InO and TKIs, which are toxic to endothelial cells, might have to be avoided, if possible. This is the first report of a potentially high risk of developing SOS/VOD when patients are administered a combination of InO and ponatinib. This might become a caution when a new clinical study of Ph+ ALL treatment combining InO and TKIs will be planned in the future. Further investigation of this issue is required in the future.

## Author Contributions

E.Y. designed and drafted the manuscript. S.O. provided a pathological diagnosis. Y.S. conducted the liver biopsy and interpreted the radiological images. T.K. supervised the study and provided the clinical information regarding the case. S.O., Y.S., T.S., N.M., M.K., K.I., M.S., M.M., A.K., and T.K. critically reviewed and revised the manuscript.

## Funding

The authors have nothing to report.

## Conflicts of Interest

Minoru Kanaya's spouse is an employee of AbbVie GK. Takeshi Kondo has received honoraria from Astellas Pharma, AbbVie, Amgen, Kyowa Kirin, Nippon Shinyaku, Ono Pharmaceutical, Otsuka Pharmaceutical, Novartis, Pfizer, and Bristol‐Myers Squibb, and consultancy fees from Otsuka Pharmaceutical. The other authors declare no conflicts of interest.

## Data Availability

The data that support the findings of this study are available upon request from the corresponding author, E.Y. The data are not publicly available because they contain information that can compromise the privacy of the research participants.
